# Predictors of Mortality in Hospitalized Patients With Pesticide Poisoning

**DOI:** 10.7759/cureus.41284

**Published:** 2023-07-02

**Authors:** Tushar Sontakke, Shriprakash Kalantri

**Affiliations:** 1 Department of Medicine, Jawaharlal Nehru Medical College, Datta Meghe Institute of Higher Education & Research, Wardha, IND; 2 Department of Medicine, Mahatma Gandhi Institute of Medical Sciences, Wardha, IND

**Keywords:** glasgow coma scale (gcs), international programme on chemical safety (ipcs pss), simplified acute physiology score ii (saps ii), acute physiology and chronic health evaluation (apache) ii score, pesticide poison, organophosphorus poisoning

## Abstract

Background

Organophosphorus poisoning (OPP) is a prevalent mortality rate that varies from 2% to 25% method of suicides worldwide. ICUs commonly employ various scoring systems such as the Glasgow Coma Scale (GCS), Acute Physiology and Chronic Health Evaluation II (APACHE II), Simplified Acute Physiology Score II (SAPS II), and International Programme on Chemical Safety (IPCS) Poison Severity Score (PSS) tools for risk stratification for mortality prediction scores and prognosis. This study aims to compare the predictive validity of these systems in hospitalized patients suffering from pesticide poisoning in a teaching hospital located in central India.

Methods

A prospective study design was utilized to gather relevant variables for calculating the GCS, APACHE II, SAPS II, and IPCS scales in patients affected by pesticide poisoning. Data on the administered doses of atropine and pralidoxime (PAM) were also recorded.

Results

We have identified several independent predictors of mortality among patients suffering from pesticide poisoning. The GCS (P=0.001), tracheostomy (P=0.001), APACHE II score (P=0.01), and SAPS II score (P=0.001) were all found to be significant indicators of mortality. Interestingly, the GCS demonstrated comparable predictive ability for mortality when compared to the APACHE II (0.82 (95% confidence interval (CI) 0.70 to 0.94)) and SAPS II (0.83 (95% CI 0.72 to 0.94)) scores, with no statistically significant difference (P=0.75) observed. Among the variables used in the IPCS PSS (GCS, heart rate, systolic blood pressure (BP), intubation, and pupil size), only GCS (P=0.05), and intubation (P=0.01) exhibited a significant association with mortality.

Conclusions

Our study determined that the GCS score, SAPS II, IPCS PSS, and APACHE II exhibited equal efficacy in predicting mortality. Notably, the GCS offered an added advantage due to its simplicity and minimal time requirements compared to the other scales.

## Introduction

Approximately 3 million individuals worldwide are subjected to organophosphates (OPs) annually, resulting in approximately 300,000 fatalities. In the United States, there are roughly 8,000 instances of exposure per year, with an extremely low number of fatalities. Although agricultural pesticides are the primary source of exposure, household products, such as ant and roach spray, contain OP compounds. [1]. Ingestion of OP insecticides is a frequently employed method for suicide attempts, often resulting in a high mortality rate [2]. Providing accurate prognostic information becomes crucial for healthcare providers and family members to make well-informed decisions during hospital admission. The Acute Physiology and Chronic Health Evaluation II (APACHE II) and Simplified Acute Physiology Score II (SAPS II) scoring systems are extensively utilized in critical care units to predict clinical outcomes [3]. To assess the severity of different types of poisonings, the International Programme on Chemical Safety (IPCS) Poison Severity Score (PSS) was developed [4]. However, the validity of these scales in predicting outcomes in the context of pesticide poisoning in India remains inadequately investigated [5].

The previous studies were characterized by retrospective designs, limited sample sizes, and inconsistent cutoff points employed to assess the precision of severity scales. One notable aspect to consider is whether the APACHE II score underwent recalibration before its implementation in the local population [6-8]. Consequently, there is a need for a comprehensive study to assess the predictive value of APACHE II, SAPS II, and the Glasgow Coma Scale (GCS) in determining the severity and case fatality among patients with pesticide poisoning admitted to a teaching hospital in Maharashtra. This study aims to fill this gap in knowledge by prospectively examining these scoring systems, incorporating a larger sample size, and utilizing standardized cutoff points. The outcomes of this study will contribute to a deeper comprehension of the prognosis associated with pesticide poisoning among the Indian population. Moreover, it will provide valuable insights to facilitate more precise treatment decisions, not only aimed at reducing in-hospital mortality rates but also concerning comfort measures and the potential involvement of end-of-life interventions.

## Materials and methods

Study design, setting and participants

This study prospectively gathered data from patients admitted to a rural medical institute in Maharashtra between January 2020 and December 2022 due to pesticide poisoning. The primary objective was to investigate the fluctuating rates of prediction regarding transfers to the ICU instead of direct admission to the ICU, along with a recommendation to include transfers from outside hospitals. The study included participants residing within a 100-kilometre radius of the hospital. Generally, the participants were young individuals from lower socio-economic backgrounds residing in the neighboring villages.

Study criteria (inclusion and exclusion)

Inclusion criteria: Patients with a documented history of pesticide exposure through ingestion or inhalation were included in the study.

Exclusion Criteria: Patients found to have ingested a non-OP compound or who died before data collection could occur were excluded from the study.

Ethics consideration and sample size

The Institutional Review Board Mahatma Gandhi Institute of Medical Sciences, Wardha, MGIMS/IEC/MED/89, approved the study protocol. The ethics committee determined that since the data collection involved hospitalized patients with pesticide poisoning who were receiving standard care and no additional interventions were being performed, the requirement for obtaining consent could be waived. The sample size calculation used Stata's "st power cox" command. Based on an estimated 200 pesticide-poisoned patients, including 20 deaths (resulting in a 10% in-hospital mortality rate), the study was projected to have 80% power to detect a hazard ratio of 1.5, with a type 1 error rate of 0.05.

Data collection

We meticulously collected data on various variables to understand the patient's condition comprehensively. Demographic information such as age, sex, residence, and the interval between the incident and arrival at the hospital were recorded. Additionally, we documented pre-hospital variables, including the estimated amount and type of poison, whether gastric lavage was performed, and the administration of atropine and pralidoxime (PAM). We also noted the presence or absence of muscarinic and nicotinic signs and any central nervous or peripheral nervous system effects. Furthermore, we collected the necessary data to compute the GCS, APACHE II score, SAPS II, and IPCS scales. Unfortunately, we could not record the levels of butyrylcholinesterase or acetylcholinesterase activity due to the limited capabilities of the hospital's biochemistry laboratory.

Patient treatment and management

Supervised by hospital consultants, residents treated the ICU patients. Our goal was to achieve rapid atropinization within 30 minutes by initiating intravenous atropine at a dose of 1-2 mg and doubling the dose every five minutes until specific targets were reached: heart rate > 80 beats per minute, systolic blood pressure (BP) > 80 mmHg, and clear breath sounds. Once these targets were achieved, an atropine infusion was initiated at 20% of the bolus dose. The attending consultants determined the frequency and dosage of PAM administration individually. Patients with respiratory failure were intubated and mechanically ventilated.

Predictors of mortality

To assess the predictors of mortality, we utilized the IPCS PSS, which was calculated based on neurological variables such as the GCS score and presence of seizures, as well as respiratory variables, including intubation, and cardiac variables, including bradycardia, tachycardia, and hypotension [9]. Additionally, we computed the APACHE II score using the necessary variables [10]. The SAPS II was also compiled using 12 physiological parameters and three disease-related variables [10].

Data analysis

For statistical analysis, we employed Stata version 13 (Stata Corporation, College Station, USA). We used appropriate tests, such as the chi-square or Fisher exact test for categorical variables and the t-test or Mann-Whitney test for continuous variables. A p-value of less than 0.05 was considered statistically significant. Cox proportional hazards models were utilized to predict in-hospital mortality, and hazard ratio estimates were calculated. Receiver operating characteristic (ROC) curves were generated to assess the sensitivity, specificity, likelihood, and odds ratios (ORs), and the area under the curve (AUC). These analyses allowed us to gain insights into the accuracy and predictive ability of the variables and scoring systems about inpatient mortality.

## Results

Table 1 shows the demographic characteristics of participants at the time of admission. A total of 217 individuals were included in the analysis. The average age of survivors was 31.9, while non-survivors had a slightly higher average age of 39.2 (P=0.02). Our study had a male preponderance, with 72.7% of survivors and 78.9% of non-survivors male. There were no significant differences in gender distribution between the two groups (P=0.55). Regarding education, the highest proportion in both groups had secondary education. Marital status showed no significant differences between survivors and non-survivors (P=0.73). Regarding occupation, laborers and farmers were the most common in both groups. The majority of participants resided in rural areas. The direct and referred type of admission and the type of pesticide ingested did not differ significantly between survivors and non-survivors. The median amount of poison ingested was 50 ml for both groups, and the median ingestion admission interval was similar. Overall, these demographic characteristics provide an overview of the participants during admission.

**Table 1:** Demography Characteristics of Participants at the Time of Admission IQR: Interquartile range

Characteristic	Survivors (N=198)	Non-Survivors (N=19)	Total (N=217)	P value
Age (mean age in years)	31.9	39.2	-	0.02
Sex (%)				0.55
Male	144 (72.7)	15 (78.9)	159 (73.3)	
Female	54 (27.8)	4 (21.1)	58 (26.7)	
Education (%)				0.35
Illiterate	17 (8.9)	2 (10.5)	19 (8.6)	
Primary	42 (21.2)	7 (36.8)	49 (22.6)	
Secondary	94 (47.5)	8 (42.1)	102 (47.0)	
College	45 (20.7)	2 (10.5)	47 (21.7)	
Marital status (%)				0.73
Unmarried	63 (31.8)	6 (31.6)	69 (31.8)	
Married	129 (65.1)	13 (68.4)	142 (65.4)	
Separated	6 (3.0)	0 (0)	6 (2.8)	
Occupation (%)				
Labourer	59 (29.8)	9 (47.35)	68 (31.3)	0.59
Farmer	69 (34.8)	5 (26.3)	74 (34.1)	
Petty shop	5 (2.5)	1 (5.2)	6 (2.8)	
Housewife	23 (11.6)	2 (10.5)	25 (11.5)	
Service	13 (6.5)	1 (5.5)	14 (6.5)	
Student	29 (14.6)	1 (5.2)	30 (13.8)	
Residence (%)				
Rural	193 (97.5)	19 (100)	212 (97.7)	0.48
Urban	5 (2.5)	0 (0)	5 (2.3)	
Type of admission (%)				0.35
Direct	62 (31.3)	4 (21.1)	66 (30.4)	
Referred	136 (68.7)	15 (78.9)	151 (69.6)	
Type of pesticide (%)				0.48
Monocrotophos	48 (24.2)	6 (31.6)	54 (24.9)	
Non-monocrotophos	150 (75.8)	13 (68.4)	163 (75.0)	
Amount of poison ingested, median (IQR), ml	50 (25-100)	50 (25-100)	50 (30-100)	0.03
Ingestion admission interval, median (IQR), min	180 (120-257)	199 (138-240)	180 (120-248)	0.87

Table 2 summarizes the results of univariate and multivariate logistic regression analyses on various risk factors associated with mortality. Age was significantly associated with mortality. Sex, ingestion-admission time, and pre-hospital interventions did not show significant associations. Bronchorrhea and dilated pupils had potential associations with higher mortality. Respiratory failure, low GCS scores, tracheostomy, intermediate syndrome (IMS), higher IPCS scale grades, and higher APACHE II and SAPS II scores were all significantly associated with increased mortality. These findings highlight important risk factors related to mortality in the study population. 

**Table 2:** Univariate and Multivariate Logistic Regression Analysis CI: Confidence interval; GCS: Glasgow Coma Scale; IPCS: International Programme on Chemical Safety; APACHE: Acute Physiology and Chronic Health Evaluation; SAPS: Simplified Acute Physiology Score; PAM: Pralidoxime; IMS: Intermediate syndrome

Risk factor	No. of events	Incidence of mortality (95% CI)	Univariate analysis	Multivariate analysis
Age, years	-	-	1.03	1.00-1.07
Sex	1.40	0.44-4.42	0.56	--
Men	15/159	9.4 (5.8 to 15.1)	-	-
Women	4/58	6.9 (2.2.16.9)	-	-
Ingestion-admission time, hours	-	-	0.99	0.99-1.00
Pre-hospital lavage	2.25	0.82-6.17	0.11	-
No	6/107	5.6 (2.35-11.94)	-	-
Yes	13/110	11.8 (6.9-19.3)	-	-
Pre-hospital atropine	1.57	0.61-4.05	0.34	--
No	9/81	11.1 (5.7-20.0)	-	-
Yes	10/136	7.3 (3.9-13.1)	-	-
Pre-hospital PAM	1.50	0.40-5.56	0.54	--
No	16/192	8.3 (5.1-13.2)	-	-
Yes	3/25	12.0 (3.3-30.8)	-	-
Bronchorrhea	2.57	0.98-6.69	0.05	--
No	8/137	5.8 (2.8-11.2)	-	-
Yes	11/80	13.7 (7.7-23.1)	-	-
Pupils	2.66	0.79-8.89	0.11	--
Normal	15/195	7.7 (4.6-12.4)	-	-
Dilated	4/22	18.1 (6.7-39.1)	-	-
Respiratory failure	3.38	1.26-9.03	0.015	--
No	11/174	6.3 (3.4-11.0)	-	-
Yes	8/43	18.6 (9.5-32.9)	-	-
Heart rate (bpm)	-	-	1.01	1.00-1.03
Admission consciousness	--	--	--	--
GCS >13	3/119	2.5 (5.4-7.5)	-	-
GCS=8-13	2/53	3.8 (3.1-13.5)	1.51	0.24-9.35
GCS<8	14/45	31.1 (19.4-45.8)	17.46	4.71-64.61
Tracheostomy	9.69	2.99-31.41	0.001	--
No	13/202	12.7 (7.5-20.8)	-	-
Yes	6/15	40.0 (19.7-64.3)	-	-
IMS	6.98	2.58-18.91	0.001	3.00
No	7/159	5.9 (2.7-11.8)	-	-
Yes	12/39	30.8 (18.5-46.5)	-	-
IPCS scale reference	3/136	2.2 (0.04-6.6)	--	--
IPCS scale Grade 2	11/53	20.7 (11.8-33.6)	11.6	3.09-53.6
IPCS scale Grade 3	5/28	17.9 (7.4-36.1)	9.6	2.1-43.1
APACHE II	-	-	1.13	1.10-1.25
SAPS II	-	-	1.08	1.05-1.12

Table 3 provides a summary of the predictors of mortality. Non-survivors had significantly higher APACHE II and SAPS II scores than survivors (P=0.001). The corrected QT interval (QTc) was longer in non-survivors (P=0.02), and their GCS scores were lower (P=0.001). Non-survivors also had a higher proportion of moderate and high IPCS PSS (P=0.001); a larger percentage had GCS scores below 8 (P=0.001). These findings indicate that higher APACHE II and SAPS II scores, longer QTc intervals, lower GCS scores, and higher IPCS PSs are associated with increased mortality risk. 

**Table 3:** Predictors of Mortality IQR: Interquartile range; APACHE: Acute Physiology and Chronic Health Evaluation; SAPS: Simplified Acute Physiology Score; QTc: Corrected QT interval; GCS: Glasgow Coma Scale; IPCS: International Programme on Chemical Safety; PSS: Poison Severity Score

Characteristic	Survivors (N=198)	Non-survivors (N=19)	P value	Total (N=217)
APACHE II score	8 (4-12)	21 (14-28)	0.001	9 (4-14)
SAPS II scores, median (IQR)	19 (7-27)	44 (28-47)	0.001	18 (10-29)
QTc interval, mean (SD), sec.	0.43 (0.05)	0.46 (0.1)	0.02	0.43
GCS, median (IQR)	11 (11-15)	6 (4-11)	0.001	15 (9-15)
IPCS PSS (%)				
Low	133 (67.2)	3 (15.8)	0.001	136 (62.6)
Moderate	42 (21.2)	11 (57.9)	0.001	53 (24.4)
High	23 (11.6)	5 (26.3)	0.001	28 (13.0)
GCS category (%)				
>13	116 (58.6)	3 (15.8)	0.001	119 (54.8)
8-13	51 (25.7)	2 (10.5)	0.001	53 (24.4)
<8	31 (15.6)	14 (73.7)	0.001	45 (20.7)

Table 4 summarizes the in-hospital treatment with atropine and oximes among survivors and non-survivors. The two groups had no significant differences in the time from ingestion to lavage. Non-survivors received higher doses of atropine at different time points and had a higher total dose than survivors. The incidence of atropine toxicity was similar in both groups. The use of PAM did not differ significantly between survivors and non-survivors. However, non-survivors received a higher total dose of PAM. These findings suggest that administering atropine and PAM may impact patient outcomes in cases of poisoning. 

**Table 4:** In-Hospital Treatment with Atropine and Oximes PAM: Pralidoxime

Characteristic	Survivors (N=198)	Non-survivors (N=19)	P value	Total (N=217)
Time from ingestion to lavage (min)	70 (45-150)	60 (45-120)	0.34	70 (45-150)
Atropine 6 hr (mg)	9.6 (3.6-18.6)	19.2 (10.2-60)	0.004	10.2 (3.6-19.8)
Atropine 18 hr (mg)	14.4 (14.4-40.0)	30 (12.4-168.2)	0.008	15.6 (5.4-42.6)
Atropine 24 hr (mg)	25.5 (11.4-63)	40.8 (24.9-255.6)	0.001	28.2 (12.0-64.8)
Atropine total (mg)	41.2 (13.8-163.8)	127.2 (46.2-496)	0.012	45.2 (15.0-169.2)
Atropine toxicity (Yes, %)	46 (23.2%)	6 (31.6%)	0.41	52 (23.9%)
Atropine toxicity (No, %)	152 (76.8%)	13 (68.4%)		165 (76.0%)
PAM given (Yes, %)	97 (49.0%)	13 (68.4%)	0.10	110 (50.7%)
PAM given (No, %)	101 (51.0%)	6 (31.6%)		107 (49.3%)
PAM initial dose (g)	0 (0-2)	0 (0-2)	0.20	1 (0-2)
PAM total dose (g)	0 (0-3.2)	10.4 (2.0-28.0)	0.05	4.4 (0.5-12.8)

Table 5 provides information on interventions during the hospital stay for both survivors and non-survivors. Endotracheal intubation was performed more frequently in non-survivors compared to survivors. The use of anti-seizure drugs was higher in non-survivors. IMS and tracheostomy were more common among non-survivors. There were no significant differences in ventilation days or length of stay between the two groups. These findings suggest that certain interventions, such as endotracheal intubation and anti-seizure drug administration, may play a role in patient outcomes in cases of poisoning. 

**Table 5:** Interventions during Hospital Stay IQR: Interquartile range; IMS: Intermediate syndrome

Characteristics	Survivors (N=198)	Non-survivors (N=19)	Total (N=217)	P value
Endotracheal intubation				
Yes	61 (30.8%)	19 (100%)	80 (36.9%)	0.001
No	137 (69.2%)	0 (0)	137 (63.1%)	
Anti-seizure drug				
Yes	50 (25.5%)	10 (52.6%)	60 (27.6%)	0.01
No	148 (74.7%)	9 (47.4%)	157 (72.4%)	
IMS				
Yes	39 (19.7%)	12 (63.2%)	51 (23.5%)	0.001
No	159 (80.3%)	7 (36.8%)	166 (76.5%)	
Tracheostomy				
Yes	9 (4.5%)	6 (31.6%)	15 (7.0%)	0.001
No	189 (95.5%)	13 (68.4%)	202 (93.0%)	
Ventilation days, median (IQR)	5(3-10)	6(2-13)	0.44	5.5(3-10)
Length of stay, median (IQR)	5 (3-9)	4 (2-9)	0.47	5 (3-9)

Determinants of fatal outcome

According to the findings presented in Table 2, the univariate analysis revealed several risk factors that displayed a significant association with mortality. These risk factors include age, bronchorrhea, bronchospasm, and miosis, respiratory failure upon admission, heart rate, IMS, total PAM dose, tracheostomy, GCS, IPSS PSS scale, APACHE II, SAP II, and QTc interval.

**Figure 1:**
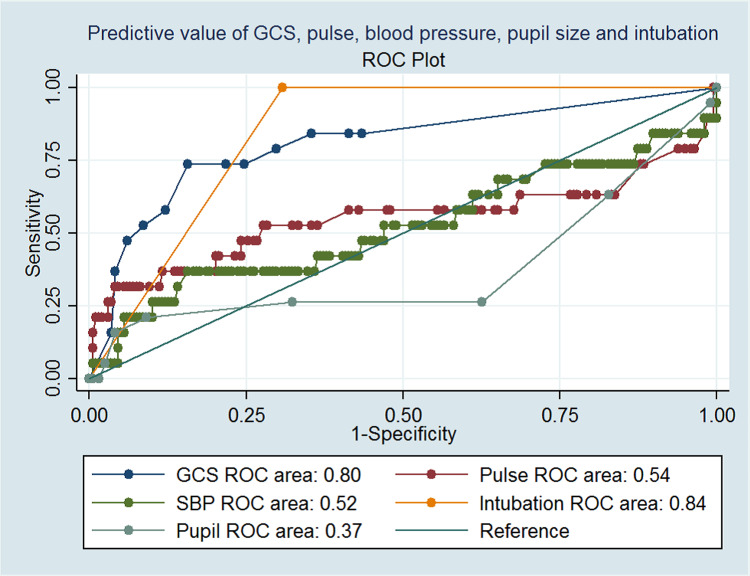
ROCs for Predicting Mortality in Pesticide Poisoning according to the Value of GCS, Heart Rate, Systolic BP, Pupil Size, and Intubation. ROC: Receiver operating curve; GCS: Glasgow Coma Scale; BP: Blood pressure

ROCs were also generated for five variables used in the IPCS PSS scale: GCS, heart rate, systolic BP, intubation, and pupil size. Among these variables, only GCS (AUC, 0.80 (95% confidence interval (CI) 0.69 to 0.94; p=0.05)) and intubation (AUC, 0.84 (95% CI 0.81 to 0.87; p= 0.01)) accurately differentiated between survivors and non-survivors, while the other three variables failed to do so effectively (Figure 1).

**Figure 2:**
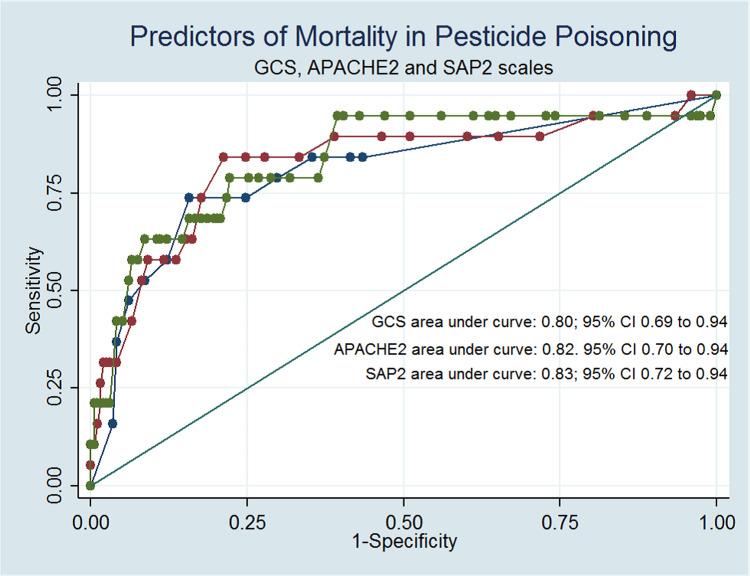
ROCs for Predicting Mortality in Pesticide Poisoning as per GCS, APACHE II and SAPS II ROC: Receiver operating curve; GCS: Glasgow Coma Scale; APACHE: Acute Physiology and Chronic Health Evaluation; SAPS: Simplified Acute Physiology Score

The GCS demonstrated comparable effectiveness to the other two scales in predicting mortality, with GCS scoring 0.80 (95% CI 0.69 to 0.92), APACHE II scoring 0.82 (95% CI 0.70 to 0.94), and SAPS II scoring 0.83 (95% CI 0.72 to 0.94). The p-value was 0.75 (Figure 2).

## Discussion

We identified several independent predictors of mortality in patients with pesticide poisoning. The GCS (P=0.001), tracheostomy (P=0.001), APACHE II score (P=0.01), and SAPS II score (P=0.001) were all found to be significant predictors of mortality. Interestingly, the GCS performed equally well as a predictor of mortality compared to the APACHE II (0.82 (95% CI 0.70 to 0.94)) and SAPS II (0.83 (95% CI 0.72 to 0.94)) scores, with no statistically significant difference (P=0.75). Among the variables used in the IPCS PSS (GCS, heart rate, systolic BP, intubation, and pupil size), only GCS (P=0.05) and intubation (P=0.01) showed a significant association with mortality. In our study, nearly a quarter of the patients had consumed monocrotophos (54 out of 217, 24.9%). Contrary to a previous study by Eddleston and colleagues, which reported high mortality associated with monocrotophos, we found that the proportion of deaths related to monocrotophos was not significantly higher (6 out of 54, 11.1%, OR 1.44; 95% CI 0.51-4.00) [11].

The median time from ingestion to admission was three hours (interquartile range (IQR): two-five hours). The median length of stay in the hospital was five days (interquartile range: three-nine days). Comparing survivors and non-survivors, there was no significant difference in the duration of hospital stay (4 days (IQR: 2-9 days) vs. 5 days (IQR: 3-9 days), P=0.47). The ingestion-to-admission time varied across different studies, ranging from 1.5 hours (IQR: one-three hours) in the Warangal study to 4.3 hours (IQR: 2.9-7.6 hours) in a Sri Lankan study and two hours (IQR: 1-1.5 hours) in a study from western Maharashtra. Despite these variations, the case fatality rates were 20.4% in the Sri Lankan study and only 1% in a group treated with high-dose PAM in the Baramati study, suggesting that the ingestion-to-admission time is not a reliable predictor of mortality. Over half of our patients received gastric lavage in primary health centers or district hospitals before arriving at our facility. All patients underwent an additional gastric lavage in the hospital after a median interval of three hours from ingestion to admission. Surprisingly, there was no significant difference in the likelihood of receiving gastric lavage between survivors (97 out of 110, 88%) and non-survivors (13 out of 110, 12%; OR 2.2 (95% CI 0.8 to 6.2)). This finding aligns with a 2009 systematic review that suggests limited evidence regarding the clinical efficacy of gastric lavage in pesticide poisoning. Current guidelines recommend performing lavage only if the patient reaches the hospital within one hour of ingestion [12].

None of the patients in our study received the recommended dose of PAM as advised by the World Health Organization or the doses used in the two randomized controlled trials [13,14]. We found no significant difference in the initial dose of PAM between survivors and non-survivors. However, there was a discrepancy in the total dose of PAM, with non-survivors receiving a higher total dose (P=0.01). More patients who died received PAM (13 out of 19, 68.4%) compared to those who survived (97 out of 198, 48.9%; P=0.10). Among the patients, a total of 108 did not receive PAM, and six of them died. Those who didn't receive PAM were older (median age 45 (IQR: 27-50)) and had a median GCS of 4.5 (IQR 4-8). It is possible that their poor GCS, rather than the lack of PAM administration, contributed to their mortality.

According to a systematic review conducted in 2011, the available evidence is insufficient to determine the efficacy and adverse effects of Oximes [15,16]. Eddleston et al., using the WHO-recommended dosage of PAM, concluded that the drug is likely ineffective and risky [17]. Two systematic reviews, which included 182 patients with OP poisoning (OPP), found no benefit of PAM [15,16]. A meta-analysis demonstrated that oximes were not associated with a reduced need for mechanical ventilation or decreased mortality in individuals with acute OPP [18]. There are as many as 38 recommendations regarding the dose of atropine in acute OPP. In our study, we followed an ICU protocol that involved administering atropine through bolus atropinization, with doubling doses over 30 minutes. Once stabilized, atropine infusion was given at approximately 20% of the initial dose and closely monitored to adjust the infusion rate.

Compared to survivors, non-survivors received higher doses of atropine within the first six hours (P=0.001), the next 18 hours (P=0.0081), the first 24 hours (P=0.0081), and throughout their entire hospital stay (P=0.01). About 40% of patients (52 out of 217) experienced atropine toxicity, with no significant difference between survivors and non-survivors (P=0.41). A third of the patients (80 out of 217, 36.9%) required endotracheal intubation, and all received mechanical ventilation for a median duration of 5.5 days (IQR 3-10). Among the 80 intubated patients, 61 (76.3%) survived, while 19 (23.7%) died. 15 patients underwent tracheostomy. Pawar et al. demonstrated that patients receiving high-dose PAM had a shorter duration of mechanical ventilation (median five days vs. 10 days) [14]. In a study by Eddleston et al., out of 376 OP-poisoned patients, 90 required intubation and ventilation, and 46 (51%) died [19].

However, we observed that patients who did not receive PAM tended to have a shorter duration of ventilation (median five days (2-10) vs. six days (IQR 3-10); P=0.14). The proportion of patients who died received a higher dose of PAM compared to survivors (P=0.004). The mortality risk was approximately seven times higher in patients with IMS during hospitalization than in those without IMS (P=0.001). However, IMS lost significance in the multivariable logistic regression model (P=0.99). We identified evidence of IMS in 51 out of 217 (24%) patients, consistent with a previous study [20]. Patients with IMS were more likely to require intubation than those without IMS (P=0.001). They also required mechanical ventilation for a significantly longer duration (median ventilator days: 8, P=0.001) and needed a higher amount of atropine (P=0.01). Indira et al. (2013) studied 176 patients and reported an incidence of IMS of 17.6% (N=31). The mean time of appearance of IMS was 44.5 +/- 22.1 hours (range: 26 hours to five days) [21]. IMS occurred in 38.7% of OPP cases and 41.9% of carbamate poisoning cases, lasting one-seven days. Neck muscles were primarily affected in patients with respiratory failure (20 out of 26). Proximal muscle weakness manifested during the later course. The emergence of IMS was associated with dimethyl OP (P=0.01) and age ≥45 (P=0.02), while multiple gastric lavages provided protection (P=0.001).

The case fatality ratio observed in our study was 8.7%, which was lower than the rates reported in a study conducted in Warangal (22.6%) and another study conducted in Sri Lanka (13% of 1,365 cases), but higher than a study from western Maharashtra (4.5% of 200 cases) [7,14,22]. Worldwide, the overall case fatality following pesticide self-poisoning is estimated to be 10%, while in India, it is around 20% [22-24]. The findings using the GCS have consistently shown strong associations with other indices of severity and outcome [25]. In a previous study, patients with a GCS score of 13 or lower had a significantly higher mortality rate (37%) compared to those with a higher GCS score (4% mortality) [7]. Two studies have compared the GCS with the APACHE II and SAPS II scoring systems [10]. The AUC, optimal sensitivities, and GCS-specificities did not differ significantly from those of APACHE II and SAPS II. However, due to small sample sizes, these studies lacked statistical power to detect even a three-fold difference in the performance of these scoring systems.

Patients with a GCS score of 13 or lower require close monitoring and aggressive treatment due to their high mortality rate. In a study by Davies et al., a GCS score of 13 or lower was the strongest predictor of mortality, with a sensitivity of 0.79. The positive predictive value (PPV) was 0.37, and the negative predictive value (NPV) was 0.96 [7]. Further analysis revealed significant differences in mortality rates among different OP pesticides. For example, in dimethoate poisoning, only 6% of deaths were missed, compared to 30% in chlorpyrifos poisoning and 56% in fenthion poisoning. Predicting mortality in fenthion poisoning is challenging because initial clinical features are delayed several days after admission [7]. Therefore, it is crucial to recognize OP pesticides as highly soluble lipid poisons that initially cause delayed effects and present with mild symptoms. In a retrospective study, the mean APACHE II, III, and SAPS II values in 48 OPP patients were reported as 11.5 ± 7.21, 42.1 ± 24.49, and 25.1 ± 15.76, respectively [26].

A small study from Karnataka, India, demonstrated that PSS scores significantly predicted mortality (p ≤ 0.001). Additionally, a significant correlation (p < 0.05) was observed between PSS, GCS, APACHE II, and predicted mortality rate scores [5]. A previous study by Bilgin et al. concluded that the GCS, APACHE II, and SAPS II were equally useful in predicting mortality rates in OPP [10]. The GCS had the advantage of being easier to perform. Another study by Grmec et al. enrolled 65 patients (including four deaths) and found that a GCS score below 6 and prolonged QTc intervals predicted the need for intubation and mortality [27]. The GCS score and the QTc interval can be used to predict respiratory failure and mortality in OPP cases. In a previous study involving 176 patients (including 50 deaths), a GCS score of 10 or lower had an AUC of 0.77, while an IPCS PSS score greater than 2 had an AUC of 0.64 [21]. A combination of GCS ≤ 10 and IPCS PSS > 2 on admission could effectively identify patients at high risk of mortality.

In our study, the GCS score and APACHE II showed similar efficacy in predicting mortality. The GCS may have advantages as it allows for data collection without additional investigations and is less time-consuming than the APACHE II system. Therefore, we suggest that the Glasgow Coma Scale is a useful tool for predicting the severity of illness in pesticide poisoning. It may substitute for the APACHE II score, as it does not provide additional predictive information compared to the GCS. It is important to note that the accuracy of the GCS in predicting outcomes may be confounded by pre-admission alcohol use and atropine treatment. Alcohol consumption is prevalent among males in our setting, and it may have additional or synergistic effects on central nervous system depression when combined with OPP. Furthermore, atropine toxicity can affect the assessment of the GCS, as many patients receive atropine in peripheral hospitals. However, we did not collect data on alcohol and pesticide co-ingestion or the amount of atropine received.

The accuracy of the GCS and IPCS PSS scores is influenced by the type of poison ingested. These scoring systems are highly discriminative for dimethoate toxicity but less precise for fenthion poisoning. In a previous study, both scales missed approximately half of the fenthion-poisoned patients who eventually died, categorizing them as low risk at presentation [7]. In the first 24 hours, a SAPS II score above 11 was found to help predict poor outcomes in OPP patients [28]. In a study conducted at Erciyes University Medical School Hospital involving 62 patients with OPP, it was observed that 26 patients exhibited a prolonged QTc interval. The mean PSS for males and females was calculated to be 1.8 +/- 1.0. Although there was no correlation between PSS and QTc intervals, a significant correlation was found between the GCS and PSS [6].

Pulse rate and pupil size were found to be unreliable predictors of mortality. Only 40 out of 216 patients in the study had a pulse rate below 60. Interestingly, those patients who did not survive had a higher pulse rate (93.7 vs. 82.0; p=0.004) than those who survived. Out of the 19 patients who eventually died, nine had received atropine before they arrived at the hospital. While some studies have proposed miosis and bradycardia as predictors of mortality, we agree with Davies and colleagues that their predictive value is limited [7,29,30]. In our study, both APACHE II (p=0.01) and SAPS II (p=0.001) were identified as independent predictors of mortality in the final multivariate logistic regression model (p=0.01). The median APACHE II score for survivors who did not require intubation was significantly lower compared to non-survivors or survivors who required mechanical ventilation (4 (1-7) vs. 17.5 (7.8-29) and 13.5 (7.8-16.3)). This study compared the modified APACHE II score with the standard APACHE II score. We found no statistically significant difference between the two systems in terms of the AUC (p=0.74) for predicting the need for mechanical ventilation [8]. GCS, APACHE II, SAPS II, and IPCS PSS demonstrated similar efficacy regarding outcome prediction. However, as a simple, bedside, and quick assessment tool incorporating physical signs, the GCS is advantageous over other scales in predicting mortality.

Strengths and limitations

Our data collection was conducted prospectively using standardized pilot-tested data collection forms to gather the information. We achieved rapid administration of atropine and initiated patients on atropine infusion following the established protocol. First, we assessed the risk factors associated with pesticide poisoning without adjusting for the specific type of pesticide (dimethoate, diethyl, or fenthion). Second, we excluded PAM from our final multivariate model since its administration was dependent on the disease's severity, which could introduce confounding factors due to indication. Third, we did not measure the activity of RBC acetylcholinesterase or plasma butyrylcholinesterase, which are essential for diagnosis [13]. However, our hospital lacked the necessary facilities to conduct such assays. Fourthly, we did not have data on patients who did not reach the hospital or those with mild poisoning. Therefore, our patient population may have presented more severe symptoms (referral bias). Finally, our study included a heterogeneous group of patients, including those with undefined pesticide poisoning.

## Conclusions

In conclusion, our study demonstrates that straightforward variables have the ability to predict in-hospital mortality in cases of pesticide poisoning. These prognostic variables provide clinicians with a clearer understanding of outcomes and facilitate more informed treatment decisions, ultimately leading to a reduction in in-hospital mortality rates. To ensure the validity and applicability of these prognostic markers, it is essential to conduct further studies involving diverse populations. Such research will contribute to the rational management of pesticide poisoning and the mitigation of mortality risks, particularly in settings with limited resources.
